# Contributing factors of elective surgical case cancellation: a retrospective cross-sectional study at a single-site hospital

**DOI:** 10.1186/s12893-017-0296-9

**Published:** 2017-09-11

**Authors:** Kaiye Yu, Xiaolei Xie, Li Luo, Renrong Gong

**Affiliations:** 1 Department of Industrial Engineering, Tsinghua University, Beijing, China; 2 Department of Industrial Engineering, Tsinghua University, Beijing, China; 3 Department of Industrial Engineering and Engineering Management, Service Management Institute, Business School of Sichuan University, Chengdu, China; 4 Operating Room Department, West China Hospital, Sichuan University, Chengdu, China

**Keywords:** Case cancellation, Contributing factors, Operating room efficiency, Quality improvement

## Abstract

**Background:**

Case cancellation (CC) has significant impact on the efficiency of operating room (OR) management, which can be mitigated by taking preventive measures. In this study, using the data of the West China Hospital (WCH), we identified the effect of contributing factors and recommended hospital interventions to facilitate CC prevention.

**Method:**

We conducted a retrospective review of 11,331 elective surgical cases from January 1 to December 31, 2014. CC reasons were grouped into six categories. The methods of descriptive statistics and hypothesis test were used to identify the effect of factors.

**Results:**

CC reasons (746) were divided into six broad categories: workup related (preoperative diagnostic assessment issues or sudden medical condition changes) (25.8%), non-specified reasons (25.8%), coordination issues (15.1%), patient related (13.0%), support system issues (11.8%), and doctor related (8.5%). The types of the most frequently performed operations are identified, as well as their CRs. The cancellation rate (CR) of males was lower than that of females (16.7% to 18.3%). A large difference in the CRs existed among doctors. The CR on Monday was significantly higher than the other four weekdays.

**Conclusions:**

Workup related issues, the types of procedures, the menstrual cycle of females, highly imbalanced CRs among doctors, and tendency of cancellation on Monday are the major identified factors, which account for a significant amount of preventable cancellations. It is suggested that corresponding hospital interventions can reduce CR and improve OR efficiency, including maintaining effective coordination, good communication and well-designed preoperative assessment processes, focusing on the type of procedures which are more time-consuming and complex, paying special attention to the physiology of females during surgery planning, taking measures to reduce CR of top eight doctors, and improving surgery scheduling on Monday.

## Background

Operating Rooms (ORs) generate the highest costs in hospitals, while also being the largest source of revenues [1]. Therefore, OR managers have substantial incentives to achieve operational excellence [2]. As one of the leading causes that decrease the efficiency of ORs, case cancellation (CC) reduces utilization of OR time, affects surgeon productivity and staff morale, causes anxiety and emotional distress in patients, and results in extra costs for patients, physicians and hospitals [3–7]. Moreover, CC also exerts burdens on society [8]. It has been reported that the lost revenue from each cancelled case is $1430 to $1700 for hospitals not on a fixed annual budget in America [8, 9].

In literature, there has been growing interest to study CRs and CC reasons by researchers. The reported CR vary widely among different countries, and hospitals of different categories. The CRs have been reported, ranging from 1% to 23% across healthcare systems in Norway, the United States, Canada, New Zealand, Great Britain, Australia, Hong Kong, and South Africa [5, 7, 9–16]. CC reasons vary significantly. Some studies have suggested that lack of available surgery resources was the common cancellation cause [10]. While others have reported that resource planning and scheduling were the major consideration [17]. Reported works have divided CC reasons into patient issues, workup related, facility reasons, planning issues, medical reasons, anesthesia related causes, and surgeon related [13, 14, 18]. While certain CC reasons are unavoidable, it has been reported that more than 50% of the CC can be prevented [11].

In the previous CC studies focusing on single centers, CRs, CC reasons, and the identified factors are reported. A hospital in Taiwan have a very low CR (0.37%) and the main CC reasons are changes in clinical condition (33.6%), the cardiovascular problem related (20.5%), the inadequate preparation (17.0%), and the surgical factors (14.8%) [19]. In an Australia referral hospital, whose CR is 11.9%, the main CC reasons include no theatre time (18.7%), no postoperative bed (18.1%), cancelled by patient (17.5%), patient clinical change (17.1%), and procedural reasons (21%), which is evenly distributed [20]. A retrospective study in a Swedish hospital over five-year period have showed that 39% of cases are canceled at least once, the major causes are patient-related issues (33%), treatment guarantee legislation issues (29%), and incomplete preoperative preparation (12%) [21]. In USA, the scholars have investigated the pediatric urology procedures cancellations in a single center and identified the preventable and nonpreventable cancellations [22]. The major preventable cancellations are insurance/financial related (11.4%) and preoperative fasting violation (8.8%), which the major nonpreventable cancellations include patient illness (40.3%) and other nonspecified reasons (29%).

In our study, using the large dataset over one-year period from a major national referral hospital in China’s western region, we identified the effect of contributing cancellation factors that has not yet been reported before through their influence on CR. We further evaluated the CC reasons and recognized preventable cancellations of elective surgeries. Also, the hospital interventions were recommended to facilitate CC prevention and improve the service level of ORs.

## Methods

### Study setting and data

After the approval of institutional review board, 11,331 surgery cases were obtained from the West China Hospital (WCH) over one-year period, 2014. The WCH is an academic and teaching hospital affiliated with Sichuan University in Chengdu, China, which is also a top class comprehensive hospital in China. It is one of the largest single-center hospital in China with 9 medical laboratory departments and 44 clinical departments, 4300 beds, and over 10,000 medical staffs. It has 99 standard ORs and 200 outpatient specialty clinics. In 2016, there are 5.3 million outpatient visits, 223,000 discharged patients whose average length of stay is 9.83 days. The annual surgery volume is 144,000. The WCH is a top referral hospital in China’s western region offering diagnoses and treatments for severe, complex, and rare diseases, which reflects a very large case mix in general. The WCH is a public hospital, supported by government funding. 50% of the patients at the WCH have medical insurance offered by government, which can cover approximately 60% of their medical cost, and the rest are self-pay patients.

In our study, all the cases were elective inpatient surgery from four specialties including cardiac, burn and plastic, neurological, and thoracic. Each case record included the date of surgery; patient ID number; name, age, and gender of the patient; department; operating room; cancellation status; and CC reason. The age of the patients varied from 2 days old to 100 years old. All the surgeries were performed on working days by 46 doctors. Normally, surgeries were scheduled before 4 PM on the day before the scheduled date. CC was defined as a scheduled surgery that was booked on the finalized OR schedule was not performed on the planned day.

More than 700 types of CC reasons were identified in the data set. We grouped them into six categories: non-specified reasons, patient related, doctor related, support system issues, coordination causes, and workup issues based on literature and the current situation of WCH [11, 13, 14, 18]. About 25% CC causes were not recorded, which were referred to as non-specified reasons. No-show of patients, sudden nonclinical condition changes of patients, temporary refusal of patients or their family, violating doctor’s advice, and other cancellations caused by patients were in the category of patient related. Doctor related reasons included the doctor being unavailable and doctors cancelling case without any reasons. The support system related reasons were associated with the issues of necessary surgery resources, including the shortage of blood, no postoperative intensive care unit beds, the OR being occupied by emergency department, malfunctioning equipment, etc. The coordination causes included the surgery information not being shared among staffs, administrative errors, and scheduling errors. Results of the preoperative diagnostic assessment should be known before the schedule was finalized, but in some cases, these were determined after the schedule was finalized, which caused the workup related cancellations. The preoperative diagnostic assessment related issues and the sudden medical condition changes of patients were the main workup causes. The categories of CC reasons were shown in Table 1.

**Table 1:** The categories of CC reasons

non-specified reasons	the CC reasons were not recorded
patient related	no-show of patients
	sudden nonclinical condition changes of patients
	temporary refusal of patients or their family
	violating doctor’s advice
	other cancellations caused by patients
doctor related	the doctor was unavailable
	doctors cancelled cases without any reasons
support system issues	the shortage of blood
	no postoperative intensive care unit beds
	the OR being occupied by an emergency case
	malfunctioning equipment
	other reasons related to resources of surgery
coordination causes	surgery information not being shared among staffs
	administrative errors
	scheduling errors
workup issues	the preoperative diagnostic assessment related issues
	the sudden medical condition changes

### Statistical analysis

We conducted statistical analysis including descriptive statistics and hypothesis test using SPSS 19.0. The CC rate was defined as the ratio of the total number of cancellations in the total number of scheduled cases. The CC rates for each gender, doctor, type of procedure and day of the week were determined. We found the relationship between the CRs and the corresponding factors including gender, doctor**,** and day of the week, respectively. Also, the ratios of cancellations by reason category were calculated. The proportions of cancellations by each category of CC reasons for doctors with exceptionally high were calculated. For each comparison, we applied the Student’s t test to compare CC rates to determine whether there were significant differences in the average CRs of all parameters [23]. It was considered to be statistically significant when the *P* value was less than 0.05. Finally, we identified and compared the preventable and unpreventable cancellations to find the preventive measures.

## Results

The overall CR was 17.5%, representing 1984 cancelled cases out of 11,331 scheduled surgeries. The 95% confidence interval was 16.9% to 18.2%, which was estimated by the normal distribution.

### Cancellation reasons

Workup related causes were the top CC reasons and accounted for 25.8% (95% confidence interval, 23.9%–27.7%) of all the cancelations, followed by non-specified reasons (25.8%), coordination causes (15.1%), patient related (13.0%), support system issues (11.8%), and doctor related (8.5%), which represented 512, 511, 299, 258, 235, and 169 cancellations, respectively. The related data were shown in Table 2.

**Table 2:** The ratios of cancellations by reason category

	Non-specified reasons	Patient related	Doctor related	Support system issues	Coordination cause	Workup related	Total
Frequency	511	258	169	235	299	512	1984
Percentage	25.8%	13.0%	8.5%	11.8%	15.1%	25.8%	100.0%
95% Confidence interval	[23.7%, 27.8%]	[11.5%, 14.5%]	[7.4%, 9.8%]	[10.4%, 13.3%]	[13.6%, 16.6%]	[23.9%, 27.7%]	–

**Fig. 1:**
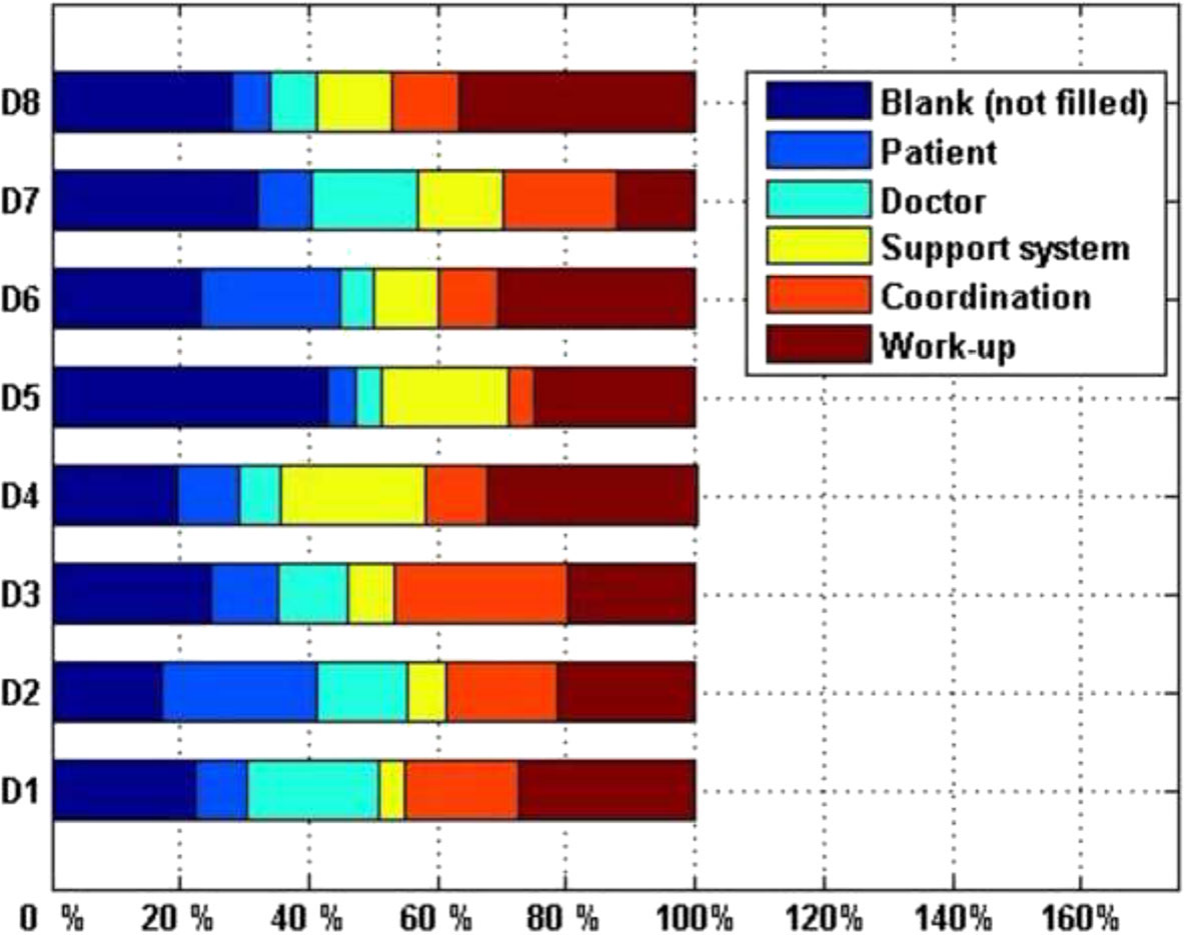
The CC reasons of the top eight doctors

Regarding the contribution of each category of CC reason to the overall CR for each of the top eight doctors, the distributions of CC reason of these doctors were not exactly same. One of the top eight doctors was from thoracic, one of them was from cardiac, and the others were from neurological. The top reasons of five doctors (D1, D4, D5, D6, and D8) were both workup related causes. However, the top reasons of the other three doctors (D2, D3, and D7) were patient related cause, coordination cause, doctor related causes respectively. The contribution of each category of CC reason to the overall CR was shown in Fig. 1.

### Cancellation rates of different types of procedures

There were 129 types of procedures in the 11,331 cases. The amount of the procedures in each type ranged from 1 to 1666. We selected ten types of procedures whose amount of cases accounted for 77.1% of all the scheduled cases. These types of procedures were resection of intracranial space occupying lesion, pulmonary lobectomy, limbs trunk injuries and deforming orthosis, cardiac valve replacement, tissue transplantation and tissue substitute material implantation and skin soft tissue expansion, clipping of intracranial aneurysm, radical resection of esophageal carcinoma, repair of atrial septal defect or ventricular septal defect, excision of intraspinal space occupying lesion, and resection of intracranial tumors, whose amounts were 1666(14.7%), 1268(11.2%), 1094(9.7%), 1073(9.5%), 783(6.9%), 780(6.9), 697(6.2%), 579(5.1%), 418(3.7%), and 383(3.4%). The distribution of the top ten types of procedures in specialties were as follows: two from burn and plastic, four from neurological, two from cardiac, and two from thoracic.

The CRs of these ten types of procedures were 39.5%(308 CCs), 23.5%(90 CCs), 20.5%(342 CCs), 16.5%(177 CCs), 16.3%(207 CCs), 15.4%(107 CCs), 12.3%(96 CCs), 12.3%(71 CCs), 11.5%(48 CCs), and 11.1%(121 CCs). The three most common cancelled operations were resection of intracranial space occupying lesion, clipping of intracranial aneurysm, and pulmonary lobectomy, whose volume were 342, 308, and 207 respectively. All above data were shown in Table 3.

**Table 3:** The related information of the top ten types of procedures with highest volume

The type of the procedure	Department	Scheduled volume	Frequency	Cancelled volume	CR of the type of cases
Resection of intracranial space occupying lesion	neurological	1666	14.7%	342	20.5%
Pulmonary lobectomy	thoracic	1268	11.2%	207	16.3%
Limbs trunk injuries and deforming orthosis	burn and plastic	1094	9.7%	121	11.1%
Cardiac valve replacement	cardiac	1073	9.5%	177	16.5%
Tissue transplantation & Tissue substitute material implantation & Skin soft tissue expansion	burn and plastic	783	6.9%	96	12.3%
Clipping of intracranial aneurysm	neurological	780	6.9%	308	39.5%
Radical resection of esophageal carcinoma	thoracic	697	6.2%	107	15.4%
Repair of atrial septal defect/ventricular septal defect	cardiac	579	5.1%	71	12.3%
Excision of intraspinal space occupying lesion	neurological	418	3.7%	48	11.5%
Resection of intracranial tumors	neurological	383	3.4%	90	23.5%

### Cancellation rate of different genders

There were 5905 scheduled cases of males and 5426 scheduled cases of females, which comprised 52.1% and 47.9% of all the scheduled cases respectively. There existed difference in average CR between males and females. The average CR of males was 16.7% (95% confidence interval, 15.8%–17.2%), representing 989 cancelled cases, and 18.3% (95% confidence interval, 17.6%–19.1%) of females, representing 995 cancelled cases. As shown in Table 4, the *P* value of T-test was 0.026, which shown that the average CR of females was significantly higher than that of males under the confidence level 0.95.

**Table 4:** CRs of different gender

Gender	Ratio of the amount	Cancellation rate	95% Confidence interval	*P*-value of T-test
Male	52.1%	16.7%	[15.8%, 17.2%]	0.026
Female	47.9%	18.3%	[17.6%, 19.1%]	

### Cancellation rate of different doctors

The 46 doctors were from four specialties: 11 from burn and plastic, 17 from neurological, 10 from cardiac, and 8 from thoracic. The mean number of scheduled cases for doctors was 246.33. High variation in CRs existed among doctors, ranging from 0 to 53.3%. We picked out eight doctors whose amount of scheduled cases exceeded or approximately equaled the mean, while their CRs was more than 20% to study the effect of doctors on cancellations. They are referred to as the top eight doctors (D1, D2, …, D8 in Table 5).

**Table 5:** CRs of the top eight doctors

Doctors	D1	D2	D3	D4	D5	D6	D7	D8	Overall
Scheduled cases	381	333	428	229	508	352	389	321	11,331
Cancellation rate	44.9%	40.2%	28.5%	27.1%	23.8%	22.2%	21.6%	21.2%	17.5%

The top eight doctors contributed significantly to the overall CR. Their respective CRs were 44.9% (out of 381 scheduled cases), 40.2% (out of 333 scheduled cases), 28.5% (out of 428 scheduled cases), 27.1% (out of 229 scheduled cases), 23.8% (out of 508 scheduled cases), 22.2% (out of 352 scheduled cases), 21.6% (out of 389 scheduled cases), and 21.2% (out of 321 scheduled cases), while the overall CR was 17.5%, as shown in Table 3. The average CR of the top eight doctors who performed 2100 surgeries (22.5% of overall performed surgeries) and cancelled 841 surgeries (42.4% of overall cancelled surgeries) was 28.6%. The average CR was 13.6% of the remaining 38 doctors who performed 7246 surgeries (77.5% of overall performed surgeries) and cancelled 1144 surgeries (57.6% of overall cancelled surgeries), as shown in Table 6.

**Table 6:** Comparison of CRs between the top eight doctors and the remaining doctors

	Number of doctors	Cancellation rate	Percentage of performed	Percentage of cancelled
The top eight doctors	8	28.6%	22.5%	42.4%
Remaining doctors	38	13.6%	77.5%	57.6%
Total	46	17.5%	100%	100%

### Cancellation rate on different days of the week

**Fig. 2:**
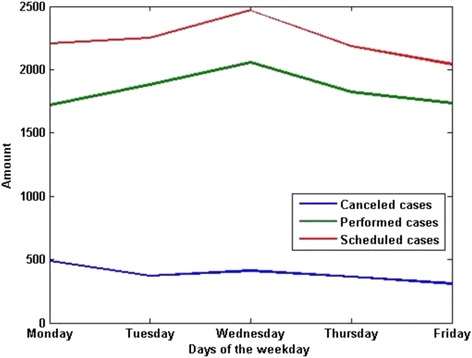
The amount of surgeries on each weekday

**Fig. 3:**
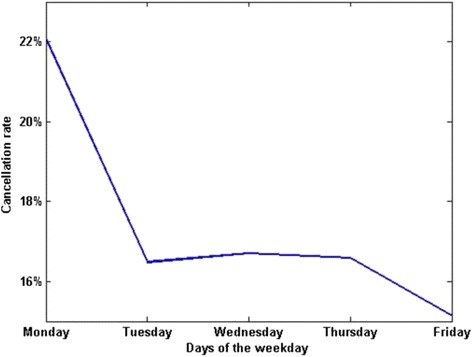
The CRs on each weekday

The CRs varied on different days of the week. The amount of performed surgeries was 1718, 1880, 2055, 1821, and 1733 respectively from Monday to Friday. The amount of performed surgeries on each day of the week was statistically same (Fig. 2). The average CRs were 22.1%, 16.5%, 16.7%, 16.6%, and 15.13% respectively from Monday to Friday. The number of surgeries and the CR on each day were shown respectively in Figs. 2 and 3. We conducted T-test to compare the CR of different days of the week, as shown in Table 7. The *P* value among the five days of the week was less than 0.05, the *P* value among four days of Tuesday to Friday was 0.474, and the *P* values between Monday and each other four days were both less than 0.05, which showed that the average CR on Monday was significantly higher than other four days, and the average CR on each day of other four days had no statistical difference.

**Table 7:** Results from the T-test of CRs of different days of the week

T-test	Monday to Friday	Tuesday to Friday	Monday and Tuesday	Monday and Wednesday	Monday and Thursday	Monday and Friday
*P*-value	.000	.474	.000	.000	.000	.000

## Discussion

### Workup related issues

The most common reason for CC was workup related, which comprised 25.8% of all cancellations, which was followed by coordination causes at 15.1%. The preoperative diagnostic assessment related issues and the sudden medical condition changes of patients were the main workup causes. Thus, more attention should be paid to preoperative diagnostic assessment related issues, sudden medical condition changes and coordination issues during the surgery management process. To avoid the top reason of CC, certain strategies should be adopted in surgery management, including well-designed preoperative process to reduce cancellations caused by preoperative diagnostic assessment related issues, information sharing among the related staffs and good communication to mitigate the CCs owing to consideration related issues.

Currently, there is not a documented pathway or program for preoperative assessment in the WCH. Before adding a patient to the operating room (OR) schedule, routine physicals and chemical examination are performed to assess whether that patient is suitable to undergo surgery on a particular day. However, after the OR schedule is created, the surgery might be cancelled due to unexpected condition changes. In the future, it is suggested to take measures to enhance preoperative assessment to mitigate its impact on CC.

### Analyses of high volume procedures

We also found out that the type of procedure was another identified factor related to CCs. We observed that the types of procedures with high volume and CRs were relatively more time-consuming and more complex, which helped explain their cancellations. Such types of procedures should require special attention.

Besides the general factors including the workup related issues and the type of procedure discussed above, we further investigate local factors including the gender, the doctor, and the day of the week below.

### Effect of menstrual cycle of females

The results demonstrated that the number of scheduled surgeries of males was higher than that of females (5905 to 5426) and the average CR of males was statistically less than that of females (16.7% to 18.3%). It was found that the case was more likely to be cancelled when the patient was female rather than male. We analyzed the reasons and consulted the staff of WCH. We found out that a significant number of cases of females were cancelled due to menstrual cycle of females. Thus, doctors or administrators should pay special attention to the physiology of females when scheduling surgery. The surgeries of females should not be scheduled when they were during their menstrual cycles.

### Highly imbalanced cancellation rates among doctors

We observed highly imbalanced CRs among doctors from the results. The 17.4% of doctors performed 22.5% of surgeries but they cancelled 42.4% of CC, which showed that the top eight doctors significantly contributed to the CC. The overall CR would decrease from 17.5% to 13.6% (a 22.3% relative difference) if the scheduled cases of the eight doctors were removed from the overall cases. In addition, the distributions of CC reasons and the top reasons were also different among the top eight doctors. The hospital administrators should focus on these doctors, investigate the reason for their high CRs, and provide corresponding solutions to address such problem. Also, different suggestions regarding CC prevention should be offered to different doctors according to their top reasons.

### Monday effect

Another observation was the so-called Monday effect, which meant the CR on Monday was significantly higher than the other days of the week, according to the results of CR comparison of different days of the week. The backlog of administrative affairs and the fatigue of staff on Mondays after the weekend led to the Monday effect. The staff of the surgery department should make an effort to improve surgery scheduling of Mondays. The number of surgeries on Mondays might need to be decreased properly to reduce the workload of the staff. In addition, the staff was supposed to keep a good weekend break to be well prepared for the Mondays.

Comparing to other single-center studies, the CR at the WCH is relatively high, especially compared to [19]. Unlike [21], we do not observe cancellation of a surgery for multiple times. Moreover, we have a large proportion of CC due to non-specific reasons, similar to [22]. On the other hand, we were also able to provide some new findings which, the best of our knowledge, have not been reported in previous literature, such as Monday effect, which help scheduling the workloads on different days in a week. The patient factor related to CC has been previously reported, however the menstrual cycle is a really intriguing factor which helps explain significant difference in CC between females and males. Another intriguing uncovered fact is that several surgeons contribute to a large proportion of cancellation.

Our study had four primary limitations. First, the data was manually recorded by the hospital staff which was observational and retrospective, resulting in bias in recording and coding of cases. Second, the study only focused on the elective inpatient surgery without consideration of the outpatient surgery and the day of surgery. Therefore, our results should be only used as a reference for elective inpatient surgical CC. Third, the investigation of cases performed only by four departments of an academic and teaching hospital, which might not be generalizable to cancellations in other types of hospital. The final limitation was related to CC reasons. The reasons of 25.8% CCs were not recorded, referred to as non-specified reasons. Thus, the CC reasons distribution may not be accurate.

## Conclusions

In conclusion, workup related issues, the types of procedures, the menstrual cycle of females, highly imbalanced CRs among doctors, and tendency of cancellation on Monday are the major identified factors, which cause a significant number of preventable cancellations. Reducing CRs and improving the efficiency of ORs require considering all the factors. We recommend hospital interventions to facilitate reducing or even eliminating the preventable cancellation. Specifically, certain strategies should be adopted during surgery management process, including well-designed preoperative diagnostic assessment process to reduce CCs caused by preoperative diagnostic assessment related issues and information sharing among the related staffs, effective coordination and good communication to mitigate the CCs caused by consideration issues. The types of procedures with high volume and CRs are relatively more time-consuming and more complex. The administrator should pay special attention to such types of procedures. Doctors or administrators should pay special attention to the physiology of females during surgery planning. The surgeries of females should not be scheduled when they are during their menstrual cycles. The minority of doctors with high CRs who perform lots of cases should be focused on when scheduling surgery. The hospital administrators should investigate the reasons for high CRs of these doctors and provide corresponding solutions to address the problem. Different suggestions regarding CC prevention should be offered to each doctor according to their top reasons. Also, the administrator should make an effort to improve surgery scheduling on Mondays. The number of surgeries on Mondays may need to be decreased properly to reduce the workload of the staff. In addition, the staff is supposed to keep a good weekend break to be well prepared for the Mondays.

## Abbreviations

CC: Case cancellation
CR: Cancellation rate
OR: Operating room
WCH: West China Hospital
